# Interaction of enamel matrix proteins with human periodontal ligament cells

**DOI:** 10.1007/s00784-015-1510-8

**Published:** 2015-07-01

**Authors:** Harsh D. Amin, Irwin Olsen, Jonathan Knowles, Michel Dard, Nikolaos Donos

**Affiliations:** Division of Biomaterials and Tissue Engineering, UCL Eastman Dental Institute, University College London, London, UK; Periodontology Unit, UCL Eastman Dental Institute, University College London, London, UK; Department of Nanobiomedical Science & BK21 Plus NBM Global Research Center for Regenerative Medicine, Dankook University, Cheonan, 330-714 Republic of Korea; Department of Periodontology and Implant Dentistry, New York University, College of Dentistry, New York, USA

**Keywords:** EMD, Periodontal ligament cells, Intracellular trafficking, EMD uptake, Receptor-mediated endocytosis

## Abstract

**Objectives:**

It has recently been shown that enamel matrix derivative (EMD) components (Fraction C, containing <6 kDa peptides (mainly a 5.3 kDa tyrosine-rich amelogenin peptide (TRAP)), and Fraction A, containing a mixture of >6 kDa peptides (including a leucine-rich amelogenin peptide (LRAP))) differentially regulate osteogenic differentiation of periodontal ligament (PDL) cells. The present study examined whether EMD and the EMD Fractions (i) bind and internalize into PDL cells and (ii) precipitate and form insoluble complexes on PDL cells.

**Materials and methods:**

Biotin-labelled EMD/EMD Fractions were incubated with PDL cells under various different culture conditions and confocal and electron microscopies were carried out to examine the binding and intracellular trafficking of these proteins.

**Results:**

The results reported here show, for the first time, that at least some components in Fraction A and the TRAP peptide in Fraction C can bind and be internalized by human PDL cells via receptor-mediated endocytosis. In addition, Fraction A was found to form insoluble aggregate-like structures on PDL cells, whereas Fraction C was soluble in culture media.

**Conclusion:**

Soluble amelogenin isoform TRAP appears to be internalizing into a subset of PDL cells. Moreover, TRAP uptake is most likely controlled by receptor-mediated endocytosis.

**Clinical relevance:**

Information on interaction between PDL cells and EMD/TRAP might prove useful in designing targeted interventions (i.e. use of chemically prepared soluble amelogenin peptides) to repair/regenerate periodontal tissues. Such interventions can also (i) avoid the use of rather crude animal-derived enamel matrix protein (EMP)/EMD preparation and (ii) preparation of cost-effective and more controlled chemically synthesized amelogenin peptides for the clinical use.

**Electronic supplementary material:**

The online version of this article (doi:10.1007/s00784-015-1510-8) contains supplementary material, which is available to authorized users.

## Introduction

Enamel matrix protein (EMP) is a heterogeneous mixture of mainly amelogenin-derived proteins produced during tooth development. Previous studies in our laboratory have shown that a heat-treated form of EMP, enamel matrix derivative (EMD), has the ability to modulate mesenchymal and non-mesenchymal differentiation pathways of adult human periodontal ligament (PDL) cells [1–4]. Moreover, certain fractions derived from EMD (Fraction C containing <6 kDa peptides (mainly a 5.3 kDa tyrosine-rich amelogenin peptide (TRAP)) and Fraction A containing a mixture of >6 kDa peptides (including a leucine-rich amelogenin peptide (LRAP), sheathlin proteins and full-length amelogenin), separated from EMD by industrial scale protein fractionation methodologies [5]), have been reported to differentially regulate a number of PDL cell differentiation activities, including osteogenesis, vasculogenesis and angiogenesis in vitro [1, 3, 4]. Although the mechanism(s) involved are not yet clear, studies of mouse amelogenins and amelogenin isoforms have shown that they can bind to and become internalized via the lysosomal-associated membrane protein-1 (LAMP-1) and can regulate dentine, cementum and PDL formation in murine embryonic dental follicle cells [6–11]. Further, some component(s) present in EMD (comprising amelogenins, sheathlins and enamelins (Brookes et al. 1995; Hu et al. 1997), as noted above) may be internalized into osteoblasts via clathrin-coated pits [12]. These observations suggest a pivotal role of receptor-mediated endocytosis in the uptake of ‘soluble’ EMP/EMD components [7, 9, 10], although the initial binding to the osteoblast cell membrane and intracellular transport and fate and the specific component(s) involved are not yet known [12].

In addition, EMD has also been observed to precipitate on human PDL tissue sections ex vivo, forming spheres or short rod-like structures [13], and also on the surface of PDL cells in vitro, stimulating cell proliferation, ECM production and osteogenic differentiation [13–15]. These data suggest the possibility that such ‘insoluble’ complexes of the full-length hydrophobic amelogenin protein, a main component of EMD, may be at least partially responsible for some of the effects on PDL cell activity [13–15].

To clarify the interaction between the commercial heat-treated preparation of EMD and the EMD fractions with PDL cells, experiments were carried out to determine whether EMD and the EMD Fractions (C and A) (i) bind to PDL cells and are internalized and transported intracellularly and (ii) precipitate and form insoluble complexes on PDL cells.

## Materials and methods

### Isolation and culture of primary human PDL cells

PDL cells were obtained from PDL tissue of patients undergoing routine third molar tooth extractions, as previously described [3]. Informed consent was in accordance with the protocol approved by the Joint Research and Ethics Committee of the Eastman Dental Institute and Hospital. The cells were cultured in growth medium (GM) comprising α-modified Eagle’s medium (α-MEM) (Gibco Life Technologies Ltd, Paisley, UK) and 10 % fetal calf serum (FCS) (PAA Laboratories, Yeovil, UK) supplemented with 200 U/ml penicillin, 200 μg/ml streptomycin and 2 mM l-glutamine (all from Gibco) at 37 °C in a humidified atmosphere of 5 % CO_2_ in air. Three separate PDL cell populations from three different donors (male, aged between 18 and 25) were used between passages 3 and 6.

### Treatment of cells with EMD components

Biotin-labelled EMD and the EMD-derived <6 kDa (Fraction C) and >6 kDa (Fraction A) sub-fractions (provided by Institut Straumann, Basel, Switzerland) were diluted in 0.1 % acetic acid, and 10 μg/ml (a concentration which has been shown to modulate a number of differentiation pathways [1, 3, 4]) was added directly to the PDL cultures at 50–60 % confluence, as described below. In addition, a chemically synthesized biotin-labelled 45-amino acid (tyrosine-rich amelogenin peptide; TRAP) peptide sequence

$$ \left[{\mathrm{NH}}_2\mathrm{MPLPPHPGHPGYINFSYEVLTPLKWYQNMIRHPYTSYGYEPMGGW}\hbox{--} \mathrm{COOH}\right] $$

provided by Institut Straumann), the major component of Fraction C, was diluted in cell culture grade water (Thermo Scientific, Basingstoke, UK) and added to the cultures at 10 μg/ml, based on previous results [3].

### Binding and intracellular uptake of EMD and the EMD Fractions by PDL cells

To assess cell binding, PDL cells were seeded onto 24-well plates on glass coverslips at a density of 10^4^ cells/well and cultured in GM for 2 days. At approximately 50–60 % confluence, the medium was replaced with cold GM containing 10 μg/ml of EMD, Fraction C or Fraction A that had been biotin-labelled. After 45 min at 4 °C, the cells were washed with cold phosphate-buffered saline (PBS), fixed with 4 % paraformaldehyde (PFA) for 15 min, reacted with Alexa Fluor-streptavidin (Cat#S-11226, Invitrogen, Paisley, UK) for 30 min, washed, overlaid with mounting medium and glass coverslips and visualized under the fluorescence microscope. To determine whether the biotin-labelled EMD and the EMD Fractions were membrane-associated, replicate cultures previously incubated with EMD and the EMD fractions at 4 °C were washed, treated with enzyme-free dissociation solution (Invitrogen) at 4 °C for 10 min, scrapped using a cell scrapper, fixed with 4 % PFA for 15 min and detached cells centrifuged at 1500 rpm for 5 min. Cell pellets were washed and resuspended in PBS, reacted with Alexa Fluor-streptavidin for 30 min, washed, air-dried on a glass slide and visualized under the fluorescence microscope as described above.

To examine whether EMD and the EMD Fractions were internalized after the initial 45 min of incubation at 4 °C as above, the cells were washed, incubated in GM at 37 °C for 3 and 6 h, fixed with 4 % PFA, permeabilized using 0.1 % Triton-X for 10 min and reacted with Alexa Fluor-streptavidin (Invitrogen) for 30 min. To ensure that any positive staining observed (i.e. red fluorescence) after incubation at 37 °C was not due to membrane-bound/extracellular ligand, replicate cultures (incubated for 3 h at 37 °C) were treated with trypsin-EDTA for 5 min prior to PFA fixation. The detached cells were centrifuged at 1500 rpm for 5 min, re-cultured for a further period of 3 h at 37 °C to allow the cells to re-adhere and spread, fixed with 4 % PFA, permeabilized and reacted with Alexa Fluor-streptavidin for 30 min as above. Nuclei were stained blue using Hoechst dye.

### Intracellular localization of EMD and the EMD Fractions

After binding, the biotinylated EMD, Fraction C and Fraction A were co-localized with markers of the trans-Golgi network (TGN) and lysosomes using specific antibodies as follows. The cells were seeded and cultured for 2 days on glass coverslips in 24-well plates and then incubated with the biotin-labelled ligands at 4 °C for 45 min. The cells were then washed, incubated in GM at 37 °C for 45 min and 3 h, fixed with 4 % PFA and permeabilized for 10 min. These time points were used because a number of ligands (mouse amelogenin isoforms, Wnt and non-toxic β-subunit of Shiga toxin) have been shown to be transported to the TGN after 30 to 60 min and then to the lysosomes after 2 to 6 h of incubation at 37 °C following binding to the cell surface [7, 16, 17]. Thus, PDL samples were immunostained for the TGN marker 58K (localized in the perinuclear region) [18]) after 45 min and for LAMP-1, a membrane protein primarily associated with lysosomal organelles [19], after 3 h, as described previously [20]. After treating with a blocking solution containing 10 % normal goat serum (NGS) in PBS for 1 h, the cells were incubated for 1 h at RT with the primary mouse monoclonal anti-58K (Sigma) (for the samples incubated at 37 °C for 45 min) and mouse monoclonal anti-LAMP-1 (Abcam) (for the samples incubated at 37 °C for 3 h) antibodies diluted 1:200 and 1:20, respectively, in PBS containing 1 % NGS. Incubation was then carried out with goat anti-mouse Alexa Fluor 488 secondary antibody (green fluorescent) (Invitrogen) diluted 1:200 in PBS containing 1 % NGS for 1 h at RT. Cells treated with non-specific mouse IgG were used as a control. The TGN was visualized by perinuclear green fluorescent staining after treatment with anti-58K and lysosomal vesicles visualized by punctate green fluorescent staining after treatment with LAMP-1. The cells were then further reacted with Alexa Fluor-streptavidin 594 (red fluorescent) (Invitrogen, Paisley, UK) for 30 min to visualize the biotin-labelled EMD and the EMD Fractions; nuclei were stained blue using Hoechst dye.

### Phase-contrast and SEM analysis of EMD, Fraction C and Fraction A interaction with PDL cells

EMD has previously been shown to precipitate and form spheres and short rod-like structures on the PDL cell surface in vitro and on PDL tissue section ex vivo [13], suggesting that that the full-length proline-rich hydrophobic amelogenin protein, a main component of EMD, might be involved in the formation of such structures [13–15]. To examine whether the TRAP-containing Fraction C and Fraction A also precipitate on the PDL cell surface, 10^4^ cells/well were seeded onto coverslips in 24-well plates and cultured for 2–3 days in GM. EMD, Fraction C and Fraction A were then added (all at 30 μg/ml), and the cells incubated for 24 h at 37 °C and examined by phase-contrast microscopy for the possible formation of insoluble particulate material. Replicate cultures were fixed in 1 % glutaraldehyde at 4 °C for 30 min and prepared for SEM analysis, using non-treated PDL cells as a control. To examine whether precipitation is possible on tissue culture plastic in the absence of any cells, 30 μg/ml EMD and the EMD Fractions in GM were incubated on cover slips for 24 h at 37 °C, fixed in 1 % glutaraldehyde at 4 °C for 30 min and examined by SEM.

## Results

### Binding and uptake of EMD and EMD Fractions by PDL cells

**Fig. 1:**
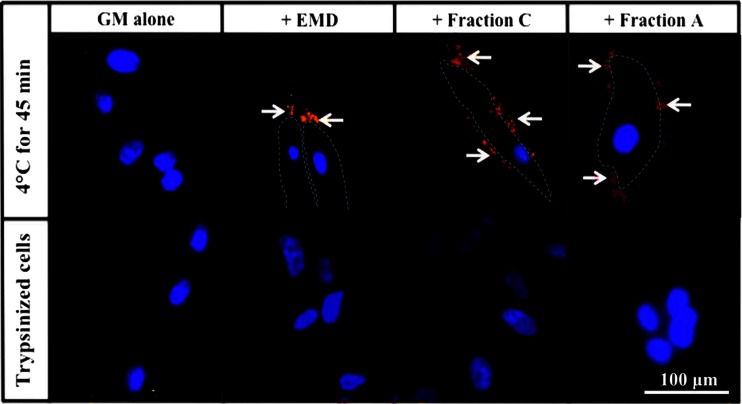
Micrographs of biotinylated EMD, Fraction C and Fraction A binding to PDL cells. Cells were cultured in the absence and presence of EMD and the EMD fractions at 4 °C for 45 min, washed, fixed and reacted with red fluorescent Alexa Fluor-streptavidin. A replicate control culture of cells incubated in GM alone was washed, detached using enzyme-free dissociation solution, fixed, centrifuged, reacted with red fluorescent Alexa Flour-streptavidin and air-dried on glass slide. EMD and the EMD Fractions are visualized by red fluorescent staining. The nuclei are stained blue using Hoechst dye. *White arrows* show cell-associated EMD, Fraction C and Fraction A. Note the absence of red staining in the control GM alone culture

The results in Fig. 1 show that, under conditions suitable for protein binding described in “Materials and methods”, red fluorescent staining corresponding to EMD, Fraction C and Fraction A was observed to be localized primarily at the cell membrane with little if any fluorescence present intracellularly. Under control conditions where cells were treated with dissociation solution post binding, there was no red fluorescent staining, indicating that EMD and the EMD Fractions were digested/removed by the trypsin/EDTA treatment and thus likely to have been mainly membrane-bound after incubation at 4 °C.

**Fig. 2:**
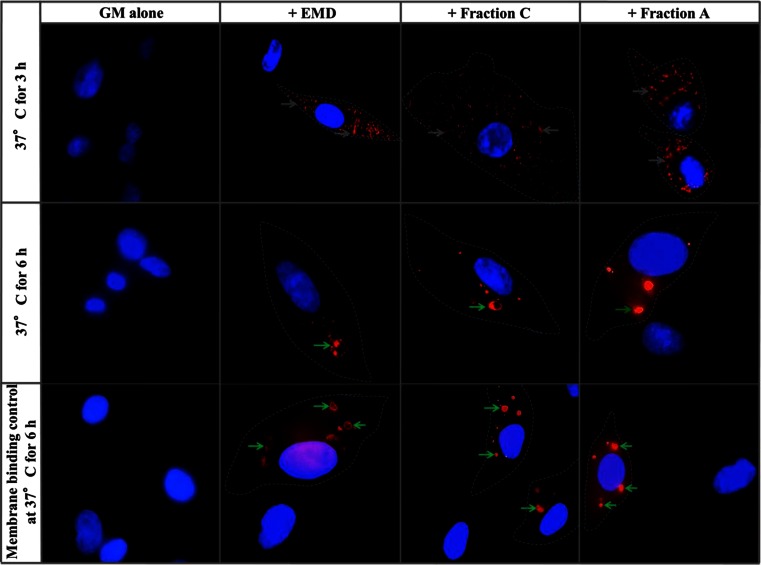
Micrographs of the internalization of biotinylated EMD, Fraction C and Fraction A by PDL cells. Cells were incubated at 4 °C for 45 min in the absence and presence of EMD, Fraction C and Fraction A, then washed and further incubated at 37 °C for 3 and 6 h, washed, fixed and reacted with Alexa Fluor-streptavidin. Replicate cultures were trypsinized to digest membrane-bound ligands and further cultured for 3 h to allow the cells to re-attach and then reacted with red fluorescent Alexa Fluor-streptavidin. EMD and the EMD Fractions are visualized by red fluorescent staining in the PDL cells. Note the *white arrows* showing the diffuse distribution of the red fluorescent EMD and the EMD Fractions. *Green arrows* show the localization of EMD and the EMD Fractions within distinct intracellular vesicle-like structures. The nuclei are stained blue using Hoechst dye

When cells cultured with the biotinylated EMD and EMD Fractions were then incubated for 3 h (protein internalization protocol as described in “Materials and methods”), the red fluorescence was localized intracellularly, with a relatively diffuse cytosolic distribution (Fig. 2). However, after 6 h, the red fluorescence was localized mainly within a few (generally <10) large, round intracellular vesicle-like structures. In control cultures of cells treated with trypsin-EDTA (post-3 h internalization protocol described in “Materials and methods”), the red fluorescent staining was still clearly evident within large, round vesicle-like structures, indicating that EMD and the EMD Fractions were internalized after incubation at 37 °C for 3 and 6 h. Manual counting of intracellular red fluorescent staining after 6 h showed that 8.5, 6.6 and 12.3 % to the total PDL cells had internalized EMD, Fraction C and Fraction A, respectively (Supplementary Figure 1).

### Intracellular localization of EMD and the EMD Fractions in PDL cells

**Fig. 3:**
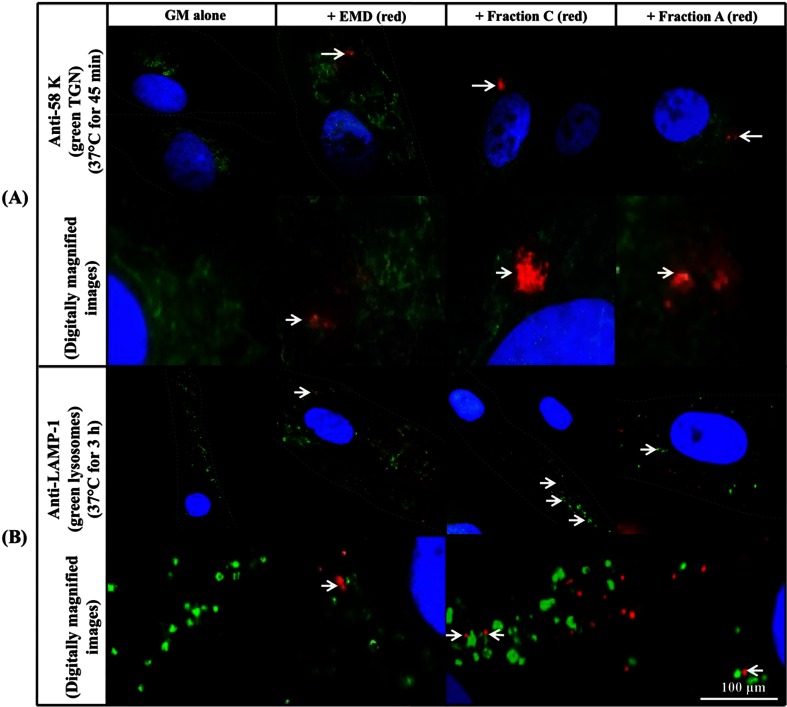
Micrographs of co-labelled PDL cells, incubated with biotinylated EMD, Fraction C and Fraction A (observed by red fluorescent staining) and immunostained for either **a** TGN marker 58K or **b** lysosome marker LAMP-1 (both observed by green fluorescent staining). PDL cells were cultured in the absence and presence of EMD and the EMD Fractions at 4 °C for 45 min, washed and re-cultured at 37 °C for 45 min (**a**) or 3 h (**b**). *White arrows* show co-localization of EMD and the EMD Fractions with 58K-positive TGN (**a**) and LAMP-1-positive structures (**b**). Nuclei are stained blue using Hoechst dye

Control cultures incubated in GM alone (with no EMD or EMD Fractions) showed no red fluorescent staining, while 58K and LAMP-1 (markers of the TGN and lysosomes, respectively) staining was observed (in replicate samples) by the perinuclear green fluorescence of the 58K antigen and the punctate green fluorescence of the LAMP-1 (lysosomal) antigen, respectively, as shown previously (Tajika et al. 2004; Bashour et al. 1998; Chen et al. 1985) (Fig. 3). The results showed that there was a substantial number (generally >50) of LAMP-1-positive diffuse structures, most probably lysosomes, as previously reported [7, 19] (Fig. 3).

**Fig. 4:**
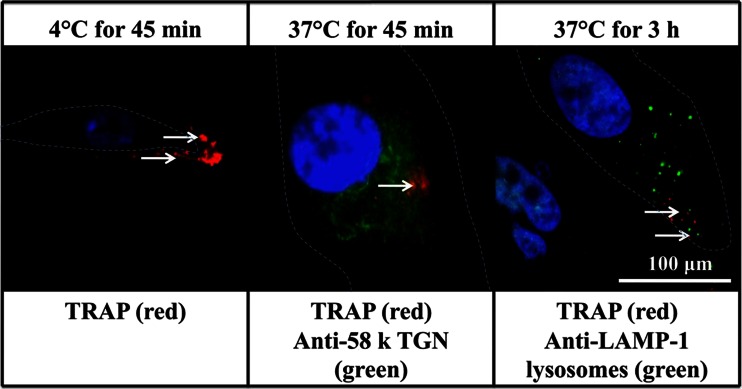
Micrographs of PDL cells incubated with biotinylated TRAP (*red fluorescent*) and co-labelled for intracellular vesicles (TGN and lysosomes; *green fluorescence*). PDL cells were cultured in the absence and presence of TRAP at 4 °C for 45 min, washed and further incubated in GM at 37 °C for 45 min and 3 h. *White arrows* show the co-localization of TRAP with 58K-positive TGN and LAMP-1-positive lysosomes-like structures. *White boxed area* shows magnified micrographs of co-localized TRAP with 58K-positive and LAMP-1-positive structures. The nuclei are stained blue using Hoechst dye

When biotinylated EMD and EMD Fractions were incubated with PDL cells at 4 °C for 45 min, washed, re-cultured in GM at 37 °C for 45 min, stained for the TGN antigen 58K (Alexa Fluor 488; green fluorescent) and then reacted with streptavidin-Alexa Fluor 594, the red fluorescent staining corresponding to EMD and the EMD Fractions was found to co-localize mainly with the perinuclear green fluorescence of the TGN (Fig. 3). However, when the incubation of the PDL cells was extended for 3 h at 37 °C, EMD, Fraction C and Fraction A were found to be co-localized primarily with LAMP-1-positive green fluorescent diffuse structures, presumably lysosomes (Fig. 3). Notably, the binding and intracellular localization of the biotinylated chemically synthesized TRAP peptide were found to be very similar to those of the EMD/EMD Fractions (Fig. 4), indicating that the intracellular fate of this synthetic peptide is likely to be similar to that of EMD, Fraction A and Fraction C.

### Formation of EMD, Fraction C and Fraction A precipitates

**Fig. 5:**
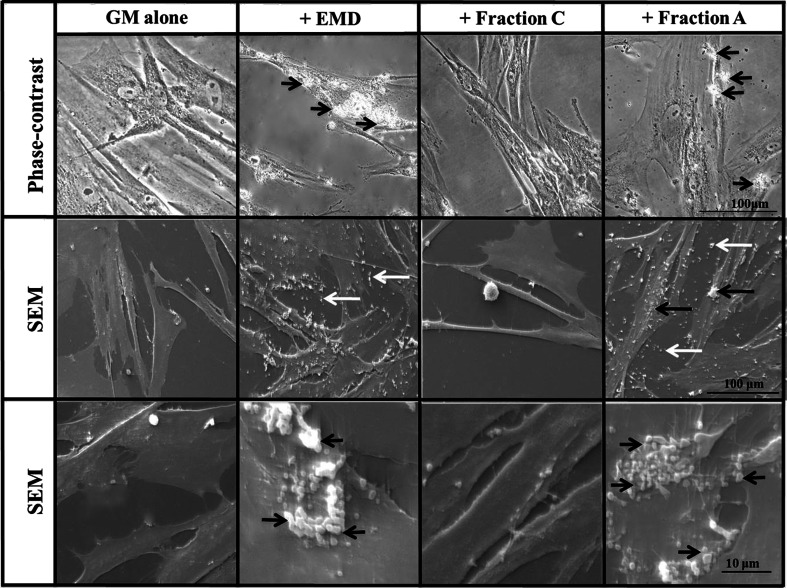
Phase-contrast and SEM micrographs of PDL cells cultured in GM in the absence and presence of EMD, fraction C and fraction A (30 μg/ml) at 37 °C. *Black arrows* indicate globular-like structures on the PDL cell surface after 24 h. *White arrows* indicate globular-like structures of EMD and fraction A directly on the tissue culture plastic after 24 h. Note the absence of globular-like structures in cultures treated with fraction C

Although EMD, Fraction C and Fraction A appeared to be readily soluble in 0.1 % acetic acid (pH 4.1), based on the clear appearance of these solutions, both EMD and Fraction A formed globular precipitates when incubated with GM alone (pH 7.4) in tissue culture plastic at 37 °C for 24 h (in the absence of any cells), as shown by phase-contrast microscopy and SEM (Supplementary Figure 2). In contrast, the low-molecular weight Fraction C did not appear to form any type of precipitate on the plastic surface (Supplementary Figure 2). When added to cultures of PDL cells for 24 h at 37 °C, both EMD and Fraction A formed spherical, aggregate-like structures that appeared to be closely associated with the cell surface and also on the tissue culture plastic surface, whereas no particulate material was detected following incubation with Fraction C (Fig. 5). Thus, while EMD and Fraction A precipitated on both the PDL cell surface as well the plastic surface after incubation in GM, for 24 h (but not by 3 or 6 h; data not shown), Fraction C produced no visible evidence of precipitation at any time examined.

## Discussion

EMD has been reported to induce PDL cell attachment, proliferation and multi-lineage differentiation in vitro and periodontal tissue regeneration in vivo [14, 21–26], although it is not yet clear whether and which components of commercially prepared (heat-treated) porcine EMD bind to specific transmembrane receptor(s) and potentiate PDL cell proliferation and differentiation. However, previous reports examining the role of mouse amelogenins in tooth development demonstrated that the full-length murine recombinant amelogenin (M180) and its isoforms (M50) and (M73) bind to the receptor CD107a (also known as LAMP-1) and to the receptor CD63 (also known as LAMP-3) on mouse embryonic dental follicle cell membranes and transduce signals for odontogenic differentiation [7, 9, 11], indicating that certain component(s) within EMD may have an important signalling role in the process of cell differentiation. In addition, it was reported previously that when human osteoblasts were incubated with a crude preparation of EMP at 37 °C for 3 h, protein component(s) were found to be internalized into cells via clathrin-coated pits, identified by simultaneous immunolabelling using anti-EMP and anti-AP-2, a clathrin adaptor protein which is responsible for linking the ligand-receptor complex (cargo) into clathrin-coated pits intracellularly (Reseland et al. 2006). However, in this study, the binding of EMP to the cell membrane and intracellular transport and fate of EMP were not examined [12]. Moreover, EMP is a complex mixture of various proteins including amelogenin, sheathlins and enamelin, while growth factors have also been found to be present in such non-heat-treated crude preparations [27]. There is therefore uncertainty about the specificity of the antibody raised against crude EMP and thus the specific component(s) that may have been internalized into the osteoblasts [12].

The results of the present study, in attempting to elucidate the mechanism(s) of this process, have shown for the first time that at least some component(s) of the commercial preparation of EMD and the EMD components Fraction A and Fraction C bind to the PDL cell membrane at 4 °C. At 37 °C, these proteins were subsequently internalized and transported to the perinuclear region of the cells, co-localizing with the TGN marker 58K after 45 min. After 3 h, these components were found to have a diffuse cytosolic distribution co-localizing with LAMP-1-positive lysosome-like structures. A previous study using a recombinant mouse amelogenin isoform (M50) showed that M50 peptide binds to the murine ameloblast (LS8) membrane after incubation at 4 °C for 1 h and internalizes and co-localizes with LAMP-1-positive lysosomes after further incubation for 1 h at 37 °C [7]. Similarly, a recombinant mouse amelogenin (M180) was reported to internalize into mouse osteoblasts (MC3T3) and ameloblasts (LS8) when incubated at for 1 h at 37 °C, having a diffuse cytosolic distribution and co-localizing with LAMP-1-positive vesicle-like structures [28]. The above studies were carried out using a murine tooth/enamel developmental process, compared with the present study which demonstrates the interactions between EMD (commercially used as Emdogain^®^ for periodontal regeneration) and EMD components with human PDL cells. It is also notable that, in the present study, when the heterogeneous PDL cell population used here was incubated with EMD, Fraction C and Fraction A, only a proportion of the cells bound and internalized these proteins, suggesting that there is an as yet uncharacterized subpopulation of cells within the PDL that expresses receptor(s) that mediates direct interaction with EMD and the EMD components.

During porcine development, EMP has been shown to be secreted by ameloblasts, specialized epithelial cells having unique extracellular projections mediating EMP secretion (Tome’s processes), and to precipitate on the surface of these same cells, promoting development of tooth-associated tissues [14]. The present study observed that both EMD and the higher molecular weight Fraction of EMD (Fraction A) precipitated and formed globular aggregate-like structures on the PDL cell surface as well as on tissue culture plastic. Similarly, recombinant mouse amelogenin (M180) has been reported to also form insoluble supramolecular aggregates [27], suggesting that this main components of EMD and Fraction A might be responsible for the structures observed here. However, further studies are required to determine which peptide(s) in the heterogeneous preparation of Fraction A (peptides ranging from 6 to 20 kDa) might be involved in forming aggregate-like structures and also whether the macromolecules present on the PDL surface are receptor-specific.

In contrast, although the present study found that the low-molecular weight component(s) of EMD (e.g. of Fraction C) did not precipitate and form such aggregate-like structures, this Fraction, containing mainly the 5 kDa peptide (TRAP) derived from full-length amelogenin, nevertheless has the potential to stimulate vasculogenic and angiogenic differentiation of PDL cells in vitro [1]. It is thus likely that at least Fraction C acts by a receptor-mediated endocytosis mechanism rather than by a precipitation-related process, although the role of phagocytosis cannot be entirely excluded since fibroblastic cells, which have been reported to be present in the PDL population [29], have also been shown to be capable of internalizing insoluble macromolecules via phagocytosis [30]. For example, collagen (300 kDa) is internalized by human gingival and PDL fibroblasts via ‘phagosomes’, a specialized intracellular phagocytic vesicle for intracellular breakdown [30]. Thus, while the present study did not investigate phagocytosis of EMD and Fraction A, it is nevertheless possible that insoluble aggregates of these components could be subjected to phagocytic uptake and thereby contribute to the functional activity of EMD.

## Electronic supplementary material

Supplementary Figure 1 (GIF 60 kb)

High resolution image (TIFF 784 kb)

Supplementary Figure 2 (GIF 88 kb)

High resolution image (TIFF 359 kb)

## References

[1] Amin HD, Olsen I, Knowles J, Dard M, Donos N (2014). A tyrosine-rich amelogenin peptide promotes neovasculogenesis in vitro and ex vivo. Acta Biomater.

[2] Amin HD, Olsen I, Knowles J, Donos N (2011). A procedure for identifying stem cell compartments with multi-lineage differentiation potential. Analyst.

[3] Amin HD, Olsen I, Knowles JC, Dard M, Donos N (2013). Effects of enamel matrix proteins on multi-lineage differentiation of periodontal ligament cells in vitro. Acta Biomater.

[4] Amin HD, Olsen I, Knowles JC, Donos N (2012). Differential effect of amelogenin peptides on osteogenic differentiation in vitro: identification of possible new drugs for bone repair and regeneration. Tissue Eng A.

[5] Mumulidu A, Hildebrand B, Fabi B, Hammarström L, Cochran DL, Dard M, Lemoult S (2007). Purification and analysis of a 5kDa component of enamel matrix derivative. J Chromatogr B.

[6] Heritier M (1982). Experimental induction of cementogenesis on the enamel of transplanted mouse tooth germs. Arch Oral Biol.

[7] Iacob S, Veis A (2008). Identification of the functional activity of the [A-4] amelogenin gene splice product in newborn mouse ameloblasts. Bone.

[8] Ten Cate A (1996). The role of epithelium in the development, structure and function of the tissues of tooth support. Oral Dis.

[9] Tompkins K, George A, Veis A (2006). Characterization of a mouse amelogenin [A−4]/M59 cell surface receptor. Bone.

[10] Xu J, Wang W, Kapila Y, Lotz J, Kapila S (2008). Multiple differentiation capacity of STRO-1+/CD146+ PDL mesenchymal progenitor cells. Stem Cells Dev.

[11] Zhang H, Tompkins K, Garrigues J, Snead ML, Gibson CW, Somerman MJ (2010). Full length amelogenin binds to cell surface LAMP-1 on tooth root/periodontium associated cells. Arch Oral Biol.

[12] Reseland JE, Reppe S, Larsen AM, Berner HS, Reinholt FP, Gautvik KM, Slaby I, Lyngstadaas SP (2006). The effect of enamel matrix derivative on gene expression in osteoblasts. Eur J Oral Sci.

[13] Cattaneo V, Rota C, Silvestri M, Piacentini C, Forlino A, Gallanti A, Rasperini G, Cetta G (2003). Effect of enamel matrix derivative on human periodontal fibroblasts: proliferation, morphology and root surface colonization. An in vitro study. J Periodontal Res.

[14] Gestrelius S, Andersson C, Lidström D, Hammarström L, Somerman M (1997). In vitro studies on periodontal ligament cells and enamel matrix derivative. J Clin Periodontol.

[15] Zeichner‐David M, Chen LS, Hsu Z, Reyna J, Caton J, Bringas P (2006). Amelogenin and ameloblastin show growth-factor like activity in periodontal ligament cells. Eur J Oral Sci.

[16] Cancino J, Torrealba C, Soza A, Yuseff MI, Gravotta D, Henklein P, Rodriguez-Boulan E, González A (2007). Antibody to AP1B adaptor blocks biosynthetic and recycling routes of basolateral proteins at recycling endosomes. Mol Biol Cell.

[17] Mallard F, Tang BL, Galli T, Tenza D, Saint-Pol A, Yue X, Antony C, Hong W, Goud B, Johannes L (2002). Early/recycling endosomes-to-TGN transport involves two SNARE complexes and a Rab6 isoform. J Cell Biol.

[18] Bashour A-M, Bloom GS (1998). 58K, a microtubule-binding Golgi protein, is a formiminotransferase cyclodeaminase. J Biol Chem.

[19] Chen JW, Murphy TL, Willingham MC, Pastan I, August JT (1985). Identification of two lysosomal membrane glycoproteins. J Cell Biol.

[20] Singhatanadgit W, Mordan N, Salih V, Olsen I (2008). Changes in bone morphogenetic protein receptor-IB localisation regulate osteogenic responses of human bone cells to bone morphogenetic protein-2. Int J Biochem Cell Biol.

[21] Donos N, Sculean A, Glavind L, Reich E, Karring T (2003). Wound healing of degree III furcation involvements following guided tissue regeneration and/or Emdogain®. J Clin Periodontol.

[22] Gkranias ND, Graziani F, Sculean A, Donos N (2012). Wound healing following regenerative procedures in furcation degree III defects: histomorphometric outcomes. Clin Oral Invest.

[23] Narukawa M, Suzuki N, Takayama T, Yamashita Y, Otsuka K, Ito K (2007). Enamel matrix derivative stimulates osteogenesis- and chondrogenesis-related transcription factors in C3H10T1/2 cells. Acta Biochim Biophys Sin.

[24] Schlueter SR, Carnes DL, Cochran DL (2007). In vitro effects of enamel matrix derivative on microvascular cells. J Periodontol.

[25] Van der Pauw MT, Van den Bos T, Everts V, Beertsen W (2000). Enamel matrix-derived protein stimulates attachment of periodontal ligament fibroblasts and enhances alkaline phosphatase activity and transforming growth factor β1 release of periodontal ligament and gingival fibroblasts. J Periodontol.

[26] Sculean A, Windisch P, Chiantella GC, Donos N, Brecx M, Reich E (2001). Treatment of intrabony defects with enamel matrix proteins and guided tissue regeneration. J Clin Periodontol.

[27] Fincham A, Moradian-Oldak J, Sarte P (1994). Mass-spectrographic analysis of a porcine amelogenin identifies a single phosphorylated locus. Calcif Tissue Int.

[28] Shapiro J, Wen X, Okamoto C, Wang H, Lyngstadaas S, Goldberg M, Snead M, Paine M (2007). Cellular uptake of amelogenin, and its localization to CD63, and Lamp1-positive vesicles. Cell Mol Life Sci.

[29] Kuru L, Parkar M, Griffiths G, Newman H, Olsen I (1998). Flow cytometry analysis of gingival and periodontal ligament cells. J Dent Res.

[30] Lee W, Sodek J, McCulloch C (1996). Role of integrins in regulation of collagen phagocytosis by human fibroblasts. J Cell Physiol.

