# Supplementary figures and images for: Interaction of enamel matrix proteins with human periodontal ligament cells
Source: Clin Oral Investig. 2015 Jul 1;20:339–47. doi: 10.1007/s00784-015-1510-8 (PMC4762925; doi:10.1007/s00784-015-1510-8)

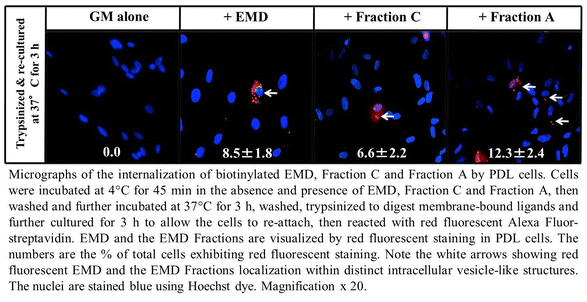

Supplement: Supplementary file 1 — (GIF 60 kb) [file 784_2015_1510_Fig6_ESM.gif]

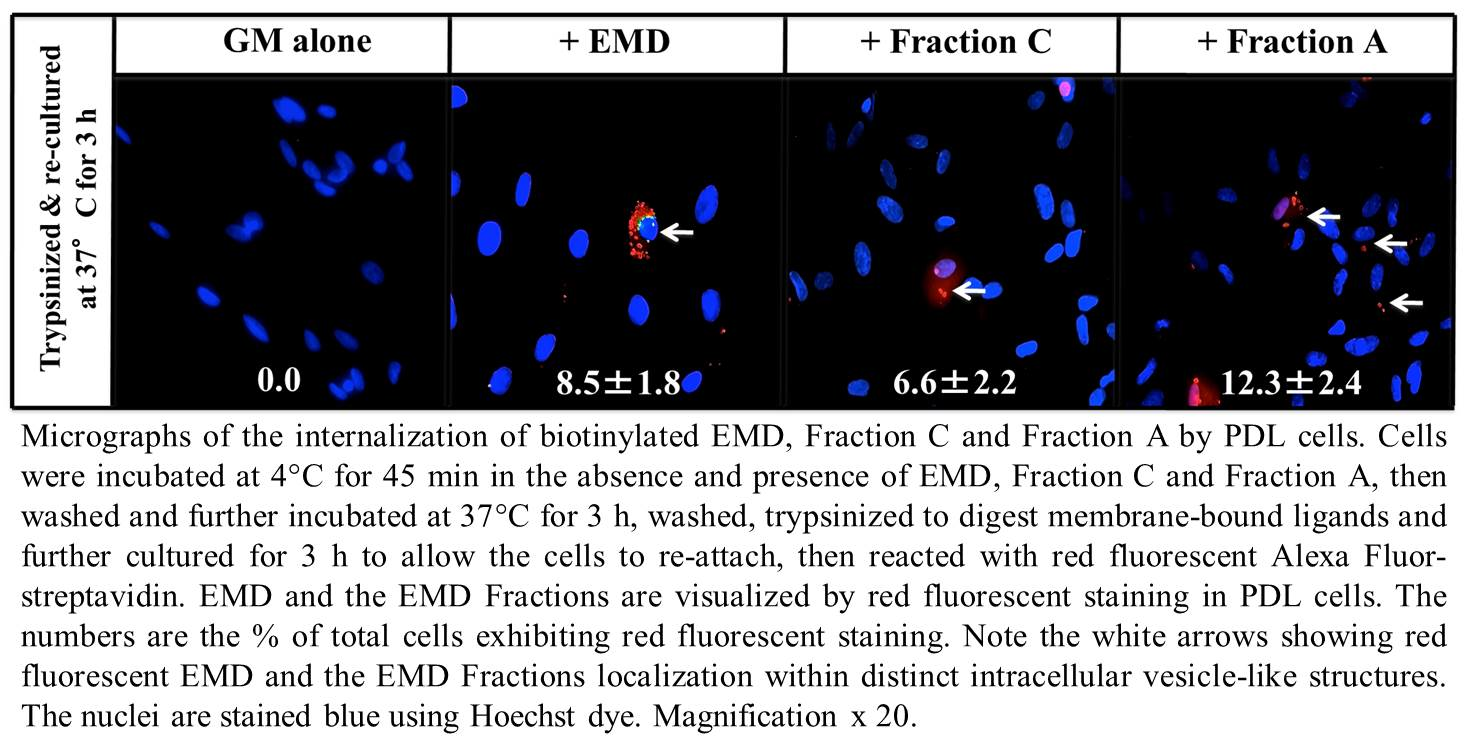

Supplement: Supplementary file 2 — High resolution image (TIFF 784 kb) [file 784_2015_1510_MOESM1_ESM.tiff]

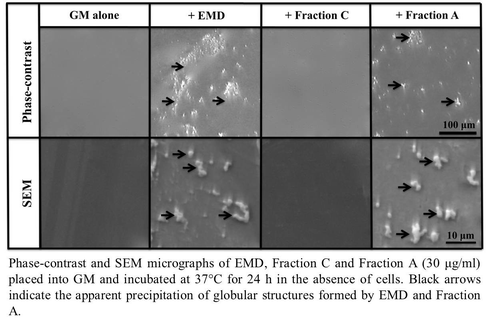

Supplement: Supplementary file 3 — (GIF 88 kb) [file 784_2015_1510_Fig7_ESM.gif]

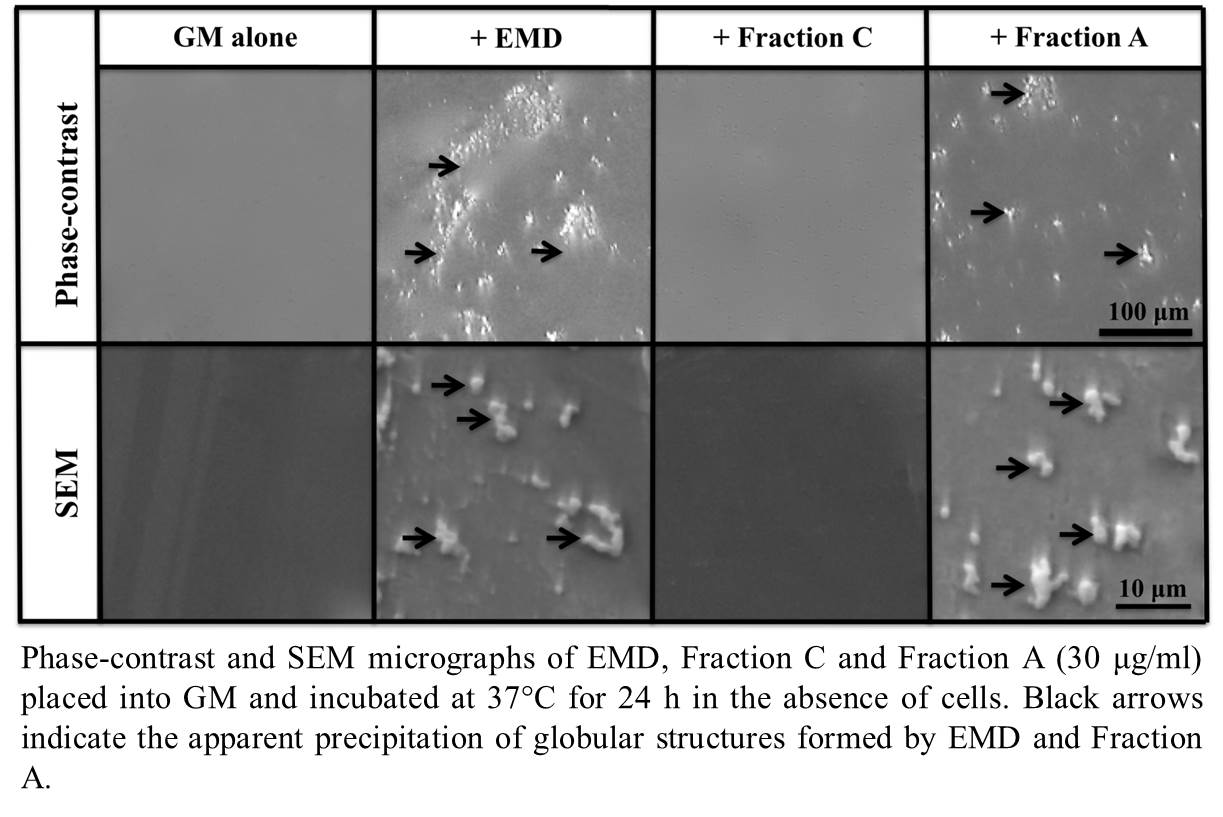

Supplement: Supplementary file 4 — High resolution image (TIFF 359 kb) [file 784_2015_1510_MOESM2_ESM.tiff]
